# Spirit of the British and American Periodicals

**Published:** 1838-04-01

**Authors:** 


### Spirit of the British and American Periodicals.

Some Observations on Abdominal Pulsation. By James Johnson, M.D.

The attention of the profession has been recently called to this subject by two papers in the July and January numbers of the Dublin Journal, by Mr. Fawssett. The second paper is entitled a "Reply to the Medico-Chirurgical Review," occasioned by some notice of Mr. Fawsset's first article, inserted at page 580 of the October number of the Med. Chir. Review. The tone which Mr. F. has assumed, and the asperity of his expressions are very unworthy of a sober and sincere inquirer after truth. I am also a little surprized that a journal which has hitherto been on the most friendly terms with this review, should have admitted some passages so outrageously disproportionate to the exceedingly slight provocation which Mr. Fawssett received from the reviewer in this periodical. But this by the way. I have not yet seen Mr. F's first paper, the Dublin Journal being mislaid, but from a perusal of the second paper, and the extracts from the first, I can form a very good idea, both of his theory and practice. From an attention of more than thirty years to abnormal abdominal pulsation, I am free to confess that the doctrine which I have adopted on this subject does not differ materially from that of Mr. Fawssett. And the same may be said respecting the practice. I think, however, that Mr. F. has taken too limited a view of the phenomenon in question. Are abnormal pulsations confined to the epigastrium—or, in more correct terms, to the aorta? Not at all. We observe them often in every tangible, or visible artery of the body. Apply the finger to the carotids in apoplexy, or cerebral congestion, and they will be felt—nay often seen, pulsating like metallic tubes. Observe the jugular veins in attacks of asthma, or in hepatization of the lungs, and *they* will be seen to pulsate. Who has not witnessed the blood spout from a brachial vein, when opened, in jets, corresponding with the abnormal pulsations of the contiguous artery? The arteries leading to a whitlow, will be felt and seen to vibrate with abnormal force and size—and the same is not unfrequently observed in a limb where gout or acute rheumatism is seated. I might adduce many other examples; but these are sufficient. My observation and reflection have led me to conclude that epigastric, as well as other abnormal pulsations depend in a great majority of cases, on one of two causes—often of both at the same time —namely, on an excited state of the heart itself, producing inordinate distention of the great arteries—or, on obstruction to the free course of blood through the capillaries of the vessels so pulsating. The two states very frequently co-exist. Epigastric pulsations so far from being rare, are extremely common. Hepatic congestion seldom exists to any extent without this phenomenon. My attention was drawn to this subject, more than 30 years ago in India, where a great proportion of men affected with hepatitis exhibited epigastric pulsation, especially when in the erect posture. This may fairly be accounted for by the turgid and congested state of the whole of the vessels in the portal circle, thus checking the discharge of blood from all the great branches of the aorta in the abdomen. The throbbings which are felt in all parts of the body suffering from congestion or inflammation, are of exactly the same nature as epigastric pulsations.

It may be objected that this phenomenon is often seen in nervous, hysterical, and hypochondriacal people, without any proof of congestion or inflammation in the abdominal viscera. I have never seen such a case, *without abnormal action of the heart itself*—a most common occurrence in the above classes of people, and clearly accounting for the epigastric pulsation. How common is it to hear nervous patients complain that they are "*pulse all over?*" And here I may observe that Mr. Fawssett has neglected to examine, or at all events, to state, the condition of the heart itself, in the four cases detailed in the January number of the Dublin Journal. I would solicit his attention to this organ in all cases of epigastric pulsation—and for this reason, that there is a visible and tangible pulsation *in epigastrio,* not dependent on, or attended by abnormal action of the aorta, but owing to the violent action of the heart itself, probably through the medium of the diaphragm, in adherent pericardium, producing a pulsation of alarming appearance at the pit of the stomach. It is hardly necessary to say that when this is owing to hypertrophy, pericardiac adhesion, or other organic disease of the central organ of the circulation, it is far more dangerous than when the cause is congestion of the abdominal vessels or viscera.

Another, and by no means unusual cause of the phenomenon in question, is a good deal *mechanical* in its *modus agendi.* I mean, constipated and loaded bowels, offering actual impediments to the course of the circulation along the aorta and its branches, and thus, not only causing turgescence of these vessels, but exciting the heart itself to increased action, in order to overcome the resistance—such epigastric pulsation is, of course, more common in females than in males, for obvious reasons.

Another cause of epigastric pulsation, and not a rare one, is irritability of the gastric nerves, so that whenever food is taken into the stomach, the heart is excited to abnormal action, and frequently accompanied by the phenomenon under consideration. I have experienced this in my own person, and have observed it in many other dyspeptics. In respect to tenderness at the epigastrium, it is to be expected in all of these cases, for obvious reasons; but this symptom is present in a great many states and conditions where there is no pulsation *in epigastrio.* I cannot persuade myself that it forms any diagnostic mark in this case.

Now if the foregoing observations be well founded—and they are certainly the result of experience, we are forced to acknowledge that epigastric pulsation is owing to several different causes—namely, congestion of the abdominal viscera—excited states of the heart itself—loaded bowels—irritability of the gastric nerves—hysteria—and perhaps other causes not yet ascertained.* Hence no one specific mode of treatment can be laid down for the complaint. If we found our pathological opinions on the therapeutical agency of medicines, we shall often build on a sandy foundation. There is scarcely a disease in Cullen's Nosology which has not been repeatedly cured (apparently) by the most opposite remedies. And, in fact, theories have been founded on the results of practice. Nothing, however, can be more fallacious. Fevers, at one time, were cured by bark and wine—and then the theory of debility, putrescency, &c. prevailed. Anon, they were cured by bleeding and purging, when, of course, inflammation was the cause of fever. The disorder or affection under consideration will often be cured by local or general bleeding, aperients, and mercurials; but we cannot safely infer from this success that the cause is invariably inflammatory or congestive. When it is caused or coupled with hepatic congestion, the symptoms of the latter are unequivocal, such as fulness of the hypochondria, sallow complexion, loaded tongue, turbid urine, depraved secretions, depressed spirits, &c. In such cases, leeching and blistering the epigastrium will assist the cure; but they are only auxiliaries. Mercurial purgatives are the sine qua non, and would, in general, remove the congestion, without any other medicine. The same treatment is necessary, when loaded bowels constitute the cause of epigastric pulsation. But if this methodus medendi were adopted in high states of gastric irritability, in nervous temperaments, and in constitutions

---

* I have noticed it in some cases of masturbation; but in these there was palpitation of the heart, a usual consequence of this depraved habit.

tinctured with the hysterical diathesis, much harm would be done. Mr. Fawssett seems to ridicule the idea of hysteria in males; but I can assure him that though men have not *uteri,* they have sometimes the disease which has been hypothetically made to depend on disordered function of that organ. It is true they will not evince fits of screaming, laughing, crying, or sobbing, like the sensitive female; but they will exhibit to the experienced eye the *hysterical diathesis,* in various grades and shades, that cannot be mistaken. In such constitutions, it is hardly necessary to say that bark and steel, and assafœtida and valerian, will be more effectual remedies than bleeding and purging, in epigastric pulsation. At the same time, even here, the secretions must be carefully attended to. In those deplorable cases where the epigastric pulsation and cardiac palpitation result from the destructive habit of masturbation, remedies have a doubtful effect. The most efficient will be found in gradually increased and long-continued exercise. Great mischief is occasionally done by mistaking these inordinate pulsations and palpitations for hypertrophy of the heart. In truth they are sometimes very difficult to discriminate from organic affections. The history, the age, and several phenomena peculiar to masturbation, are more to be depended on than auscultation.

---

### Stricture of Œsophagus, communicating with the Trachea.

Mr. Lindesay has related this melancholy case in the Calcutta Transactions, vol. 8. Serjeant Casey, aged 55, generally temperate and healthy, came into hospital, in June 1833, complaining as if his chest had struck against a hard body, which was the case. From that time he felt wandering pain in the upper part of the right side of thorax, with gradually increasing dysphagia. No disease of heart, lungs, or ribs could be detected. The pain was soon removed by leeches, blisters, and attention to the general health; but the dysphagia remained unmitigated. The smallest tube of the stomach-pump could be passed into the stomach, giving a feeling of continued resistance for an inch or two opposite the top of the sternum. When the tube was withdrawn, its orifice was always filled with puriform mucus streaked with blood. He was somewhat benefited by the occasional passings of the tube. He left the hospital in fair general health; but returned in August of the same year with increased dysphagia, having lived for some time on thin bread and milk, very slowly swallowed. Two ineffectual attempts with the tube were made; a third attempt, with an elastic catheter, and with considerable force, succeeded in passing the stricture. Next day, a weak solution of lunar caustic was ingeniously applied to the stricture by means of a sponge fixed on the point of the stilette of the catheter, and made to protrude through the eye of the instrument at the part. No inconvenience followed, and the operation was repeated next day, and for several days afterwards, but with no decided benefit. On the 2d of October, the plan recommended by Mr. Fletcher, of Gloucester, was tried—namely, the introduction of a piece of flaccid gut, which was then inflated and drawn back. It receded easily, but did no good. On the 26th of November the poor fellow applied again to Mr. L. He was then troubled with cough, and hawked up glairy mucus with blood. In a violent fit of coughing he now threw up a mass of coagulated blood, and afterwards could swallow nothing. On introducing a little fluid *to* the part strictured, it was coughed up from the trachea, proving the communication between the two passages. He is now much emaciated. Nutritive enemata. 27th. Has not swallowed a drop since yesterday. Retained the enemata for several hours. To be repeated. Warm bath. 28th. Retained the enemata pretty well. No deglutition. Tried the tube, and got it through with difficulty—poured down wine and water. Thinks the enemata do much good. The tube was a second time passed, and nutriment thrown in. He lingered till the 14th of December, when death put an end to his sufferings.

The upper portions of the lungs were much congested and infiltrated—the rest healthy, as was the heart. An opening into the trachea from the œsophagus, irregularly circular, the size of a shilling, was found, situated midway between the cricoid cartilage and bifurcation. The mucous membrane of the trachea, for some distance above and below the aperture, was vascular, and covered with purulent mucus. The œsophagus was not diseased, until just above the bifurcation of the trachea, where it was found thickened and ulcerated in its whole circumference, the ulcer occupying full four inches of the canal, and of very irregular surface, with almost cartilaginous projections.

We believe that most practitioners will acknowledge that œsophageal strictures seldom admit of much benefit from mechanical dilatation. We verily believe that more harm than good is done by bougies in such cases. We have seen more relief from constant counter-irritation to the external surface—nourishment by enemata—and the liquor potassæ, with iodine—than from any other means. We have not noticed any external application in the above case. People may be nourished much longer than is supposed by broths and beef-tea, with laudanum, *per rectum*.

---

Obscure Disease resembling the "Grand Climacteric." By Dr. Johnson.

A gentleman, when about 60 years of age, began to complain of a weight, not pain, in the umbilical region, and consulted several of the most eminent medical men in London—among others, Sir A. Cooper, and Sir B. Brodie. Neither these nor any others could detect any tumour, tenderness, or sign of structural affection. His appetite continued good—he was rather corpulent; and he was in the habit of eating meat suppers, and taking his wine and brandy and water as usual till within three months of his death. Five years ago he became affected with a most obstinate singultus which lasted nearly a fortnight, night and day. He was then attended by the late Mr. Walker, of Piccadilly, and Dr. Johnson saw him for the first time. The singultus gave way to large doses of musk, and Dr. J. examined Mr. S——— very carefully in search of the complaint which had annoyed him for some years, but was unable to detect the slightest deviation from the normal condition of parts. The feeling of weight and discomfort, however, continued, and Dr. J. did not attend Mr. S. again till the beginning of July, 1837, when the patient presented a remarkable alteration in his physiognomy — namely, that which characterizes the "climacteric disease." His appetite had fallen off—he had lost flesh, but was not at all emaciated—his tongue was white and his pulse was quicker than natural. The evacuations, however, were healthy—he had no pain on pressing every part of the abdomen—but the old complaint of "weight in the umbilical region" was his daily theme. Various means were tried to restore the appetite, but with no success. He grew feebler and feebler—had some nausea—and in August had two or three paroxysms resembling ague, which came on with rigor at a certain hour, and ended in a profuse perspiration. The old attack of hiccup returned, when the attacks of ague went off, and lasted nearly a week. Still he had no pain in any part of the body, but he declined daily in strength and flesh, while the nausea and disinclination for food increased. A few days before his death (in the latter end of September) Dr. Seymour examined him very carefully with Dr. Johnson, but could detect no organic disease. He gradually sank exhausted in the 67th year of his age.

The body was examined by Mr. H. J. Johnson, in presence of Dr. Robert Lee and Dr. Johnson, when the following appearances were presented.

On opening the abdomen, the stomach and intestines were generally distended with flatus, and in most parts of a dark slate colour. Their appearance was that of an advanced stage of putrefaction, although less than twenty-four hours had elapsed since his death.

The termination of the transverse arch of the colon, its descending arch and sigmoïd flexure, and some coils of small intestine which were contiguous to these portions of the great, were matted more or less together, and unnaturally fixed in the left lumbar and iliac fossæ. The adhesions were not of a very recent character.

The intestinal canal was laid open through a considerable part of its extent.

The mucous membrane of the stomach and duodenum was pale, soft, and in a half dissolved state. This appeared rather to result from a putrefactive than a morbid process.

The descending colon, and especially its sigmoïd flexure were so fragile that the slightest touch ruptured them. All the coats appeared to be thickened, and the mucous was remarkably pulpy and soft. There was reason to believe that there existed an aperture in the external and posterior wall of the colon at its sigmoïd flexure by which the interior of the bowel communicated with a large and sloughy abscess in the left iliac and lumbar fossæ.

This abscess was of considerable extent, reaching from the kidney to the ligament of Poupart. The psoas muscle was almost totally destroyed, and sloughs of its fibres and of cellular membrane resembling macerated tow were found in some quantity in the cavity. The latter was circumscribed in front, by the colon and its adhesions to surrounding parts—and behind it had almost arrived at the skin of the left loin. Its contents were the sloughs to which allusion has been made, and a very imperfect sort of pus. The term sloughy abscess is scarcely calculated to convey an idea of the condition of the parts, but it would be difficult to find another more appropriate. The affection was evidently of a chronic character.

The gall-bladder contained a large number of calculi, which had all the features of merely inspissated bile.

No other disease of consequence was discoverable. The head was not examined.

Remarks.—The foregoing case offers a remarkable illustration of the amount of organic change that may exist in the human frame without corresponding symptoms. No reference was ever made by the patient to the region where the disorganizing process was going on—nor were there any indications of gall-stones at any period for many years before death. Indeed it is not probable that any of the biliary calculi ever got into the ductus communis at all, and therefore, no pain, sickness, or jaundice were liable to occur. It is, however, exceedingly likely that the attacks of obstinate singultus were owing to the accumulation of gall-stones—and perhaps the same observation might apply to the sense of weight, or, as the patient always characterized it, "load" in the umbilical region. This grievance was complained of for several years, and therefore may be supposed to have a cause of anterior date to the lumbar disease.

---

### Hepatic Abscess bursting into the Colon and into the Lungs, with Recovery. By Dr. Colledge.—

[*From the Calcutta Quarterly Medical Journal.*]

Such fortunate results as the following are so rare that they deserve to be put upon record, as holding out the signal of hope when the darkness of despair is gathering around.

"*Case of Mr. J. C. S.*—He was seized on the 6th of August, in Canton, with inflammatory symptoms, for which he was leeched on the 10th or 12th, and bled from the arm on the 14th or 15th, and had this treatment afterwards followed up by a succession of leeching and blistering, and the administration of calomel every night, until the severity of the symptoms gave way. The disease was so far got under before his leaving Canton, that he was considered out of danger by his medical attendants, and was recommended by them to go to Macao for the benefit of a purer atmosphere, where he arrived on the 1st of September, labouring under a relapse of all his former symptoms, but of an aggravated and more strongly marked character. He complained of much acute tenderness over the whole region of the liver; so much so, as to be scarcely able to bear any degree of pressure of the hand upon any part of it. An attempt even to take a deep inspiration caused very severe pain in the right side. His respiration was short, quick, and attended with cough; tongue coated, mouth parched; quick and sharp pulse: anxiety of countenance, and great general prostration; symptoms clearly indicating that the inflammatory process had exceeded *the bounds* which admit of a termination of active disease by *resolution*.

The application of leeches to the seat of pain, which was had recourse to *repeatedly*, and carried as far each time as his reduced state would admit of, afforded only temporary relief. His bowels were carefully attended to and kept open by means of emollient clysters, with occasional small doses of calomel, and rhubarb and castor-oil.—Counter-irritation by means of blisters and the tartar emetic ointment was kept up;—the nitro-muriatic bath was tried, and persevered in for some time; notwithstanding all which, no decided benefit was produced.

The above treatment was pursued until the 13th, when a sudden change for the better, in the character of the symptoms, took place. He felt himself all at once relieved, and was sensible of something having given way within him. On examining his motions next day, a very considerable quantity of purulent matter was discerned in them, and in those he passed for several days after, which sufficiently warranted the opinion that had been held, of an abscess having formed in the liver.—For ten or twelve days after this, he improved considerably; when another return of the symptoms took place. The same remedies were employed as before, together with anodyne fomentations, with the same want of success; he got daily worse; and serious apprehensions regarding his recovery were entertained,—when, on the 4th of October, he experienced another sudden change for the better. But this abscess being higher situated in the organ than the former one, burst into the *thorax* instead of the *colon*, and the matter was discharged by expectoration. Ever since he has continued to get better; and nothing further was required than a careful attention to the state of the bowels,—keeping them open by mild aperients and emollient clysters,—improving the strength generally by demulcent tonics and a strictly regulated diet,—and allaying nervous irritability and procuring sleep by means of night-draughts containing the acetate of morphia.

A few days ago he felt some uneasiness in the right side: the cupping-glasses were had recourse to, but as he could not endure them, leeches were applied in their stead, and with a very good effect. He is now recovering rapidly. (Signed)

T. B. COLLEDGE.

*Macao, 22nd Nov.* 1836.

There is too much brevity in the above case. The matter expectorated should have been carefully examined; for if bile was not mixed with it, the probability, or at all events the possibility, was, that an abscess had formed in the lung itself contiguous to the liver, and was discharged by the mouth. We have seen three cases of hepatic abscess bursting into the lungs. One recovered after a long illness—the other two died.—*Editors.*

---

PHLEBITIS.

*Disease of Veins.* Dr. Monat, of Bengal, has published a paper on this subject in the eighth volume of the Calcutta Transactions, from which we shall extract the chief contents.

*Case* 1. Sergeant Smith, aged 33, in India 14 years, subject to frequent attacks of dysentery, was admitted into hospital on the 7th of March, 1834, for severe dysentery, complicated with hæmorrhoids, fever, and disorder of digestive organs. The urgent symptoms were removed in 13 days—treatment not mentioned—but he continued weak, with diarrhœa, febrile paroxysms, anorexia, dyspepsia, &c. with occasional swelling of the feet. On the 15th of September he was confined to bed, having suddenly experienced an acute pain in the right leg, from the hip to the ankle, with soreness in the line of the femoral vessels, which felt hard and painful on pressure, relieved by leeches and fomentations below the groin. On the 17th he complained of pain in the opposite leg, from the iliac region to the knee, accompanied by fever, cold perspirations, and debility. These symptoms were also removed by leeches and fomentations. On the 19th the vessels of the right leg felt hard, and painful when pressed upon. Those on the left were soft and painless. From this period he continued easy, though exceedingly weak, till the evening of the 24th September, when the pain returned with great violence in the left leg. He expired a few hours afterwards in great agony.

*Dissection.* No disease in the head or chest. Liver was slightly enlarged, and pale. Some old ulcers in the colon and rectum. The common iliac vein of the left side was filled with a firm coagulum, extending into the internal iliac as well as the external and crural veins, as far as the ham, where it gradually terminated. At the upper part the coagulum adhered to the internal surface of the vessels, which was slightly red. These veins were much distended, with a puffed hard appearance about the situation of the valves. The vessels of the right side were healthy. Both lower extremities were slightly œdematous.

We are ready to admit that the above case might come under the head of phlebitis; but we appeal to our obstetric brethren if it bears the slightest analogy to the common phlegmatia dolens of women *post partum.* The whole dispute, indeed, lies in a nutshell.—Phlegmatia dolens has been confounded with phlebitis—the former a disease hardly ever fatal—the latter very frequently so.

*Case* 2. J. Martin, a stout, muscular soldier, aged 30, and five years in India, admitted 9th August, 1834, with a severe attack of remittent fever—incessant cough—epigastric pain—giddiness—anorexia—debility, &c. "*The fever was removed by copious depletion, and a sore mouth from calomel.*"* The cough and mucous expectoration, however, continued, with relaxed bowels, which annoyed him till the 15th of September, when they left him, and in the beginning of November he was considered convalescent. On the 7th Nov. he was suddenly attacked with quotidian paroxysms of fever, preceded by rigors, acute headache, &c. These continued till the 16th, when the fever ceased, and he was once more convalescent. Next day he complained of great pain in the left shoulder—and on the 18th in the left side, in the region of the spleen, with tenderness on pressure, quick pulse, hot skin, and general restlessness Leeches removed these symptoms, till the 22d, when the pain of side returned, increased by a full inspiration, but relieved by the loss of sixteen ounces of blood from the arm. On the 25th, complained again of pain in the left shoulder and in the calf of the left leg—pyrexia being present. Venesection to 12 ounces, with great relief. 27th. The pain in both localities still more acute—relieved by leeches and fomentations. On the 1st of December he suddenly experienced great pain in the left groin, and along the femoral vessels, extending to the ankle and foot, much increased by pressure, the vessels

* How does this accord with that Star of the East, Dr. Dickson, of Cheltenham, who boasts of never wetting a lancet, or using any depletion whatever in fever or any other disease. Verily his brethren of India are most obstinate in resisting the torrents of new light which this Clarissimus Preceptor is shedding on the medical horizon!

feeling like cords, and the leg and foot swelled and pitting on pressure. Leeches to the femoral vessels below the groin gave relief. 2*d Dec.* Continued easy—leg shining and tense, but less swollen. Iliac and femoral vessels felt hard like cords, but not painful on pressure. In the evening great soreness was complained of in these parts—the limb tense and glossy, with fever. Fomentations, &c. continued. 3*d*. Appeared better; but exacerbation in the evening, with irritable stomach, and pyrexia. Leeches to the groin afforded relief. 4th. Much the same. 5th. Delirious—leg and instep much swollen, vessels hard, stomach irritable, tongue dry, great thirst. Ten ounces of blood from the arm—blister to the epigastrium. A quarter of a grain of morphia at night. 6th. Better—no pain—leg less swollen—stomach still irritable. Morphia repeated. 7th. Seemed better. 8th. Slept ill—respiration quick and laborious—leg much swollen—pulse feeble—great anxiety—tongue crusted. He died in the afternoon.

*Dissection.* No disease in the head. In the thorax were found firm adhesions between the lungs and pleura costalis, with gelatinous exudation intervening—large quantity of water in the pleural cavity and pericardium.* *Abdomen.* Stomach healthy—mucous membrane pale—same of the intestines—a large abscess in the spleen—liver diseased, the right lobe resembling Spanish soap, or red sandstone—left lobe exhibiting the white dram-drinking character.—Other viscera sound. *Left* thigh, near the groin, 21½ inches in circumference—right thigh 17 inches. Much fluid in the cellular substance of the left or diseased limb—the saphena major and superficial veins much diseased, their cavities being filled with a firm and seemingly organized secretion—deep-seated veins in the same condition. The iliac and femoral veins of the same side were enormously enlarged, and similarly diseased, this state extending into the vena cava, to an inch or two above the junction of the common iliacs. The coagulum, when examined, was firm, of a dark red colour, its surface covered with a pus-like secretion, which appearance characterized the whole venous system of that side, down to the foot. The internal tunics of the vessels were tinged red. The heart was rather large, and very flabby.

Dr. Monat considers this case as very analogous to the cases of *phlegmatia dolens* described by Dr. Davis and others. It resembles the cases of *phlebitis*, no doubt, of Drs. Davis, Lee, &c., but we still maintain that it is not the phlegmatia dolens known to accoucheurs, as by no means uncommon after parturition, and hardly ever productive of death. How is it that we never fail to have a *post mortem* after the phlegmatia dolens of Dr. Davis, but never can get such a thing after the disease as witnessed so frequently in the bed-chambers of the parturient females. This, in itself, is rather indicative of dissimilarity in the diseases. Dr. Monat seems to think that that curious and dangerous disease, the BERI BERI, is phlebitis. In the following sentiment we are inclined to agree with Dr. M.

"Perhaps future inquiries may indicate that there are two kinds of disease, separate and specific, *usually called* phlegmatia dolens."

---

### Tympanitis Abdominalis.

A German gentleman resident in London, had consulted Dr. Johnson occasionally for 14 years past, on account of a disease of the heart. The chief symptoms were dyspnœa on taking exercise—fluttering and palpitation in the region of the heart—cough, &c. This gentleman was frequently examined with the stethoscope. The case presented phenomena which are by no means rare, but which have not been described by any author in a clear manner. On listening to the actions of the heart, it would seem as if all the four chambers acted quickly in succession, instead of two and two at a

---

* We regret to find that our Indian brethren *appear* to neglect auscultation and percussion. Even in Mr. Twining's case, attended with so much obscurity, the application of the stethoscope seems never to have been dreamt of!!

time. But, in fact, the sounds were incapable of description in language, although they never could be forgotten by any one who had listened to them once. The impulsions were by no means strong, except now and then, when the apex of the heart thump'd against the parietes —and then fluttered. There was a kind of tumult heard in the region of the central organ.

In the present case swellings of the ankles were frequently observable, and the urinary secretion, as usual, defective. The patient was a free liver, and about 56 or more years of age when he died. About the middle of February 1838, Dr. Johnson was called to him, in Bedford-place, Russell-square, when he was found to be affected with symptoms of cholera, viz. violent vomiting and purging—pulse scarcely distinguishable —and general collapse. By means of opiates and cordials, these urgent symptoms were relieved, and then came on a different train of symptoms. There was pain and tenderness in the abdomen—furred tongue—thirst—quick and intermittent pulse, high-coloured urine. Leeches, fomentations, enemata, mild aperients, &c. mitigated these symptoms; but did not dissipate them. Although free and copious evacuations were daily procured, the abdomen enlarged gradually but steadily, and exhibited a true tympanitic state. The tympanitis amounted, in a few days, to a degree of distention which has been rarely seen in a thin male subject like the present. For two or three days before his death (25th February) he appeared like a female in the last month of pregnancy—the abdomen being tense and sonorous as a drum. As the alvine evacuations were freely kept up, it was evident that this was tympanitis abdominalis, and not intestinalis. Calomel and opium—mercurial frictions—and other means were used; but as the cardiac disease was still in existence, the patient sank and died.

The body was examined by Dr. Johnson and Mr. James (of Bedford-place) on Sunday the 25th of February, eight hours after death. The tympanitis was most extraordinary. The instant that the abdomen was punctured, an immense collection of air rushed forth, and the abdomen fell to nearly its natural size. There were marks of recent inflammation throughout the whole of the peritoneal cavity, evinced by recent adhesions between the convolutions of the intestines by means of soft coagulable lymph. The transverse arch of the colon, as in some cases of mania, made a bend down to the symphysis pubis. There was about half-a-pint of serum effused into the peritoneal cavity. This gentleman had been in the habit of drinking a bottle of wine, and several glasses of brandy daily; yet the liver presented no appearance of organic disease. There were evidences of inflammation in the mucous membrane of the stomach and bowels.

The lungs were sound. The heart was greatly hypertrophied, the parietes of the left ventricle being an inch in thickness. The mitral valve was so puckered and distorted that it was quite incapable of performing its office of preventing the regurgitation of blood into the auricle. In the left ventricle there was a polypus (as it used to be called) so dense in structure, and so adherent to the internal surface of the cavity, that it was impossible to separate it without great force, so as to tear it in pieces. It extended into the aorta. A similar appearance presented itself in the right ventricle.

There are two or three points of interest in this case. It would appear that the irritation and inflammation of the mucous membrane of the stomach and bowels were suddenly transferred or extended to the serous membrane of the abdomen—and that then the tympanitic symptoms commenced, and arrived at a most unusual extent. Is tympanitis abdominalis, caused by any thing else than peritoneal inflammation? The results of our own observations would lead us to answer in the negative.

The phenomena presented by the action and sounds of the heart, in this case and in analogous cases, are referable, in the narrator's opinion, to dilatation, whether active or passive, of the left ventricle, with valvular imperfection. In this case, the absence of regular impulse, corresponding with the degree of hypertrophy, may be fairly laid to the account of the imperfect state of the mitral valve, which permitting a regurgitation into the auricle, prevented the ventricle from exercising that strong action that would tell on the ribs in the shape of impulsion.

---

### DICKSONIANISM AT HOME AND ABROAD.

From a very humorous address delivered by Dr. Hayden lately, in the Peter-street School of Medicine, Dublin, we extract the following remarks on our amiable, talented, experienced, and judicious friend, Dr. Dickson, of Cheltenham. This gentleman is labouring hard for FAME—per fas aut nefas—and seems to think that, so long as *notoriety* is gained, a main object is attained. The end will justify the means! In a late number of this Journal we laughed at rather than seriously criticised the absurd doctrines which Dr. Dickson essayed to spread through the ranks of the profession, and the furious fulminations which the Indo-Cheltenham, M.D. has ever since been launching forth against the Editor of this Journal, for the innocent strictures of the Reviewer, must have been very edifying to the public, and no less gratifying to the author of the "FALLACIES OF PHYSIC." A bold Hibernian will now come in for his share of the Cheltenham anathemas, and Dr. Hayden may prepare to be buried in the bog of Allen, or drowned in his own dear Liffey.

"I think you will be out of all patience with *one* Dr. Dickson, of Imperial Square, Cheltenham, who lately in defence of HIS 'Principles of Practice of Physic,' states in the "Lancet"—a very unsuitable vehicle I should think for such STUFF—'It is now some years since I first repudiated the *Lancet* as a therapeutic agent; an instrument invented in an age of barbarism; the first and only resource of ignorant pretension in almost every case and country.'

There, gentlemen is an unlimited and sweeping exclusion of one of our most useful remedies! No wonder the public should be sceptical, when such trash is published by one yclept *a Doctor!*

It was justly observed, that true religion has been at all times, and in all ages, greatly injured by *pretenders.* The same remark holds good in reference to the practice of physic.

Berkely, the celebrated immaterialist (one who says there is no such thing as matter in the world) the bishop of tar-water notoriety, one day knocked loudly at Swift's hall-door—the witty dean ordered his servants not to open the door; thrust his head out of the window, and cried out to Berkely, 'why don't you come in out of the rain?—can't you come through the door; sure there's no such thing in nature as matter!'

God between this Cheltenham *Doctor* and all harm, (as we say in Ireland), but I should like to be at his elbow if it so happened that he was seized with an attack of Peritonitis, such as invaded the poor shoe-maker's wife; although from the sample we have of his brains, *phrenitis* might be a more probable event.

Suppose, gentlemen, the Doctor on his back, his legs drawn up, and I, an obstinate Irish fellow, *alone* and *only* at hand. The agonised patient should faithfully promise to burn all the unsold copies of HIS Practice of Physic, and make a handsome and suitable public apology to the '*repudiated lancet,*' before the much maligned instrument should afford him the relief to be obtained from it, and IT ONLY. Let us dismiss the Doctor with, *requiescat in pace.*"

But Dr. Hayden is very much mistaken if he thinks Dr. Dickson will "remain in peace." The Cheltenham Doctor would soon die if he were not at war with his brethren—and the profession itself.

From the *West* let us turn our eyes to the *East,* and see how resplendent our Cheltenham luminary shines. It is well known that one of the charges which Dr. Dickson makes against Dr. Johnson, is the mischief which the work of the latter on Tropical Climates has done to Dr. D. and his patients in India.

It was the boast, some fifty years ago, of Mr. Curtis and a few others, that they "never *wetted a lancet* in the diseases of the East"—and Dr. Dickson has carried his antiphlebotomising and antidepletory to the highest pitch of absurdity, which any man in or out of Bedlam could possibly do. Let us see how his brethren of the East countenance this MONOMANIA ANTIPHLEBOTOMICA (if we may be allowed to coin an epithet for the Cheltenham nondescript) and his torrent of abuse againt the work of Dr. Johnson on the diseases of the East. In the second Number of the INDIA QUARTERLY JOURNAL of MEDICAL SCIENCE, published at Calcutta in April 1837, a long and able retrospect of the Anglo-Asiatic practice of men for the last half-century, is given, from which we extract the following passage, for the edification of the Cheltenham luminary.

"Dr. Johnson's work is, in many respects, highly valuable. If it were only for his manfully enforcing the antiphlogistic system, and his powerful advocacy of the necessity of bleeding and purging in acute tropical affections, he would be entitled to the respect and gratitude of all Indian practitioners."—*India Quarterly Journal, April,* 1837, p. 147.

We leave Dr. Dickson to ponder on this testimony from his Indian brethren, on a work that has stood the test of twenty-five years, and gone through five editions. When Dr. Dickson's Practice, or rather fallacies of physic, has undergone such an ordeal, and received, at the expiration of a quarter of a century, such a probat from distant strangers, it will be time for him to exult—but not till then.

---

CASE OF HIDROSIS, OR HIDROTIC FEVER. BY MR. J. C. W. LEVER. 8vo. pp. 24. Barker, London.

The case was that of a very nervous lady, 36 years of age, and pregnant of her first child. Any agitation or excitement produced, habitually, headache and diarrhœa. During the last three months of utero-gestation she suffered from sickness, and also from a dull pain in her left side, near the linea semilunaris, attended with diarrhœa. She was very sleepless—pulse variable, but steadily above 100—cold perspirations. On Sunday, 7th May, she was delivered of a living child. Uterine hæmorrhage followed, owing to adherent placenta, which was carefully removed, and the hæmorrhage ceased. On the second day after confinement the pulse was 130—lochia abundant—diaphoresis —no urine passed. Catheter introduced, and 16 ounces drawn off. Third day (May 9th) nothing particular; but in the evening, there was increase of mental excitement — pulse 135 — earthy smell about the patient. Fourth day, better in all respects—pulse 110. Fifth day, at 4 in the morning, smart general rigors, attended with violent palpitation and dyspnœa, followed by profuse perspirations—dull pain in the left iliac region—lochia apparently mixed with a sanguineo-purulent matter—secretion of milk—countenance good—pulse 150 —bowels not open—urine free. Eight leeches to the painful part—three grains of calomel and half a grain of opium—followed in two hours by castor oil—febrifuge mixture.

*Sixth day.* Pulse 98—diaphoresis—lochia of the same quality—the aperient had acted plentifully. In the evening, there was increase of nervous excitement —pulse 130—profuse sweats—urine copious and pale.

*Seventh day.* Better.

*Eighth day,* better also; but in the afternoon a profuse discharge of blood from the uterus, mixed with some coagula and with pain.

*Ninth day.* Rigors, accompanied by palpitation and dyspnœa, lasting 15 minutes, and followed by profuse perspiration of strong disagreeable odour. There were pain and tumidity above the pubes. Twenty ounces of urine drawn off by the catheter, pale—pulse 150—breathing hurried — tongue clean — bowels open—lochia profuse and sanguineo-purulent. A strong opiate ordered in infusion of roses. Another rigor in the afternoon, but slighter.

*Tenth day.* Restless night—in other respects much the same—no rigors—pulse 160.

*Eleventh day.* Pulse 150—perspiration profuse—lochia still purulent. Tried the bisulphas quinæ, with mineral acids and tinct. hyoscyamus.

*Thirteenth day.* Pulse reduced to 96; but complains of pain in the head—perspirations less profuse, lochia scanty—urine free.

*Fourteenth day.* Sub-tympanitis—pulse 100—lochia scanty—tongue clean bowels open. The quinine continued. The bowels had a tendency towards diarrhœa during the next two days, scarcely checked by opiates.

*Sixteenth day.* Diarrhœa—head hot—stupor—delirium—imperfection of vision—slight subsultus—tympanitis—pulse 154—breathing hurried. Dr. Blundell saw her at 2 p. m. and ordered several medicines, including mercurial ointment to the thighs, and cold lotion to the head. Dr. B. saw her next evening (17th day) when there did not appear to be much alteration in the symptoms. The tympanitis distressing—diarrhœa.

*Eighteenth day.* She sank and expired.

DISSECTION.—*Head.* Nothing abnormal. The same might be said of the lungs, except a few calcareous deposits in the upper lobes. *Heart* flabby, with two ounces of clear pale water in the pericardium. *Abdomen.* Intestines much distended with flatus—particularly about the cæcum—peritoneal coat of intestine not shiny, but dull—stomach not injected; but the mucous membrane easily separable from the other coats—liver flabby, pale, and soft to the touch—it was, in fact, what may be called a fatty liver—kidneys flabby, pale and very destitute of blood. The lining membrane of the small intestines was not inflamed or ulcerated; but the glands of Peyer were enlarged. *Uterus.* On its posterior surface was a tubercle, the size and shape of a broad-bean, tough and firm under the knife—structure of uterus pale and firm—lining membrane of a dark piony colour—on the right side, near the fundus, was an elevated spot, the size of half-a-crown, of a dark brown colour. "Within and around this elevation the openings of the uterine veins could be seen, and, on making pressure, there was seen issuing from some of them a decided purulent fluid, from others pus mixed with blood." There were some minute ulcerations in the lining membrane of the uterus. Around some of these ulcerations, the proper substance of the uterus appeared softened and easily lacerable by the probe. A careful examination of the venous trunks, which were laid open from the internal and external surface, exposed obstruction by coagula, or a purulent fluid. In the abdominal and pelvic vessels there was nothing unusual found.

This case is a very interesting one. Looking at the *pathology* we would set it down as a case of unequivocal uterine phlebitis, or hysteritis; and we are not sure that the profuse perspirations which accompanied the uterine disease should induce us to change the name which is stamped on the malady of *post-mortem* examination. In cases of phlebitis—in the similar cases of secondary inflammations after accidents and operations, profuse sweats, and symptoms with those of the present case are equally common and characteristic.

